# Multidirectional Chromosomal Painting in the Harpy Eagle (*Harpia harpyja*): Conservation of Breakpoints in Accipitriformes

**DOI:** 10.3390/ani16050799

**Published:** 2026-03-04

**Authors:** Fábio Augusto Oliveira Silva, Rodrigo Petry Corrêa de Sousa, Anderson José Baia Gomes, Patrícia C. O’brien, Malcolm Ferguson-Smith, Edivaldo Herculano Corrêa de Oliveira

**Affiliations:** 1 Programa de Pós-graduação em Neurociências e Biologia Celular, Campus Guamá, Universidade Federal do Pará, Belém 66075-110, Pará, Brazil; faosufpa@gmail.com; 2 Instituto de Estudos Costeiros, Campus Bragança, Universidade Federal do Pará, Bragança 68600-000, Pará, Brazil; rodrigopcsousa@gmail.com; 3 Instituto Federal de Educação, Ciência e Tecnologia do Pará, Abaetetuba 68440-000, Pará, Brazil; anderson.gomes.ifpa@gmail.com; 4 Cambridge Resource Centre for Comparative Genomics, Cambridge CB3 0ES, UK; allsorter@gmail.com (P.C.O.);; 5 Seção do de Meio Ambiente, Instituto Evandro Chagas, Ananindeua 67030-000, Pará, Brazil

**Keywords:** avian cytogenomics, ancestral karyotype, chromosomal rearrangements, comparative chromosome painting, karyotype evolution

## Abstract

Diurnal birds of prey (Falconiformes and Accipitriformes) often display karyotypes that diverge markedly from the putative ancestral avian condition (2n = 80), with reduced diploid numbers and fewer microchromosome pairs driven by extensive chromosomal rearrangements. The harpy eagle (*Harpia harpyja*) was the first raptor analyzed by chromosome painting, revealing a karyotype (2n = 58) shaped by both microchromosome fusions and macrochromosome fissions followed by secondary fusions. However, these earlier analyses were limited in probe coverage. Here, we present a comprehensive chromosomal map of *H. harpyja* using multidirectional chromosome painting combined with chromosome-level genome assembly data. We integrated cross-species probes from *Gallus gallus* and *Leucopternis albicollis* with high-resolution genomic data to refine syntenic relationships and identify fission–fusion hotspots. *G. gallus* probes confirmed most previously described and genomically inferred associations but revealed novel features, including a new GGA1/GGA3 association and an increased number of GGA1-derived segments (five to six). Genomic data did not support previously suggested fusions involving GGA20–HHA1 or GGA12–Z. Dual-probe FISH further uncovered lineage-specific rearrangements, indicating rapid chromosomal evolution within Accipitriformes. This integrative approach clarifies harpy eagle genome organization and highlights dynamic evolutionary restructuring in raptor chromosomes.

## Simple Summary

Compared with most avian species, diurnal birds of prey, including hawks and eagles, frequently exhibit atypical karyotypes, with lower diploid numbers, and lower number of microchromosomes. The mechanisms and evolutionary pathways underlying these chromosomal changes remain incompletely understood. However, comparative chromosome painting has significantly advanced the understanding of avian karyotype evolution, revealing recurrent chromosomal macrochromosome fissions and fusions with microchromosomes. The harpy eagle was the first bird of prey to undergo partial comparative chromosome painting analysis using a pool containing chicken chromosome paints for pairs 1–6. The present study aimed to expand this analysis by employing the full set of available chicken chromosome painting probes (GGA1–GGA11), generating a detailed chromosomal map of the harpy eagle through the integration of chromosome painting results with high-quality chromosome-level genome sequence data. Our results confirm several previously reported chromosomal rearrangements and reveal novel structural changes, while also indicating that certain previously proposed chromosomal fusions are unlikely to have occurred. Overall, our findings demonstrate that the harpy eagle genome has undergone extensive structural reorganization during its evolutionary history. These results contribute to a deeper understanding of avian genome evolution, providing an improved cytogenomic framework to support future evolutionary studies.

## 1. Introduction

Diurnal birds of prey (Falconiformes and Accipitriformes) often display karyotypes that diverge markedly from the putative ancestral avian condition (2n = 80), with reduced diploid numbers and fewer microchromosome pairs driven by extensive chromosomal rearrangements [1,2,3]. Later, the application of comparative chromosome painting using *Gallus gallus* (GGA) probes has revealed extensive genomic reorganization in this group. Moreover, the introduction of additional sets of chromosome-specific probes from other species has added a new dimension to comparative cytogenetic studies [4,5]. Among the most informative probes are those derived from *Leucopternis albicollis*: reciprocal chromosome painting between this species and *G. gallus* demonstrated that its karyotype (2n = 66) also originated from fissions of ancestral macrochromosomes and fusions involving both microchromosomes and macrochromosomes [4]. Thus, the *L. albicollis* chromosome-specific probes correspond to defined regions of the larger *G. gallus* chromosomes, facilitating the identification of chromosomal breakpoints in other species and enabling the detection of homologies and rearrangements such as fissions and fusions.

The first species to have its karyotype analyzed using comparative chromosome painting was the harpy eagle (*Harpia harpyja* Linnaeus, 1759) [6]. This analysis revealed a chromosomal organization that differed from earlier hypotheses, which had primarily attributed the reduced diploid number observed in some species of this group to microchromosome fusions [2,3]. The harpy eagle, also known as the American harpy or royal hawk, is one of the largest and most powerful birds of prey in the world and is considered a symbol of tropical forest biodiversity. With a wingspan ranging from 1.8 to 2.2 m, it is capable of exceptional maneuverability in dense forest environments. Its talons are long and extremely robust, adapted for capturing arboreal prey such as sloths and monkeys. The plumage of the harpy is predominantly gray, with a distinctive, erectable crest that gives the species a unique appearance [7,8].

The cytogenetic analysis of *H. harpyja*, a species with 2n = 58 [6,9] using whole chromosome probes marked a turning point in the evolutionary study of birds of prey, as it was the first representative of Accipitriformes to have its karyotype characterized through comparative chromosome painting, as previously mentioned. Although early studies employed only a partial set of *G. gallus*-specific probes, the results profoundly redefined our understanding of genomic restructuring processes in this group. They demonstrated that the relatively low diploid number (2n = 58) in the harpy eagle resulted from complex evolutionary history, involving multiple fissions of ancestral chromosomes and an intricate pattern of rearrangements affecting nearly the entire genome. This has revealed a unique genomic organization among birds [6].

Despite representing a major advance in avian comparative cytogenetics, the initial chromosome painting study left significant gaps in the understanding of chromosomal rearrangements involved in the karyotypic evolution of the group. This limitation stemmed from the use of a restricted probe set, covering only pairs GGA1–6 and the GGAZ, without precise identification of macrochromosomes GGA7–10. Additionally, the hybridization quality was suboptimal, as a seven-color pool was used in place of individual chromosome-specific probes [6]. Furthermore, there was no information available regarding macrochromosome fission points, since probes from other species were not employed. Since then, additional species of Accipitriformes have been examined using various sets of whole-chromosome painting probes. To date, comparative chromosome painting data are available for 14 species of Accipitriformes, ranging from New World vultures, which exhibit karyotypes similar to that of *Gallus gallus* [10], to taxa with more derived karyotypes and reduced diploid numbers, such as Old World vultures [11] and New World buteonines [12]. Based on chromosomal characters derived from these analyses, and including two species of Falconiformes, Nie et al. [9] conducted a cladistic analysis mapped onto a consensus molecular phylogeny. Their results demonstrated that, with the exception of New World vultures (Cathartidae), the karyotypes of Accipitriformes and Falconiformes—formerly classified within a single order—have undergone extensive chromosomal reorganization through multiple fusion and fission events [13].

More recently, a chromosome-scale genome assembly of a female *H. harpyja* was published, generated using a combination of long-read sequencing, optical mapping, and chromatin conformation capture technologies [14]. These high-resolution genomic data added further complexity to the picture by suggesting an unusual translocation between an autosome and the Z sex chromosome, potentially indicating a multiple sex chromosome system [14]. However, this hypothesis has not yet been corroborated by cytogenetic evidence and appears to contradict classical cytogenetic data, which demonstrate remarkable conservation of the ZW sex chromosomes in this species and in other previously analyzed Accipitriformes. Chromosome painting results suggest that the ancestral Z segment remains largely intact, with complete hybridization observed on both the Z and a portion of the W chromosome in *H. harpyja* [6].

Therefore, the present study aims to clarify the genomic organization of *H. harpyja* by integrating multidirectional chromosome painting using chromosome-specific probes from *G. gallus* and *L. albicollis* with the available chromosome-scale genome assembly. Our primary objective is to provide independent physical cytogenetic validation of large-scale rearrangements inferred from in silico genome analyses, which can be ambiguous in complex, repetitive, or pericentromeric regions, as well as to detect and refine chromosomal fissions, fusions, and interstitial insertions that may be difficult to resolve through sequence assembly alone. By combining these complementary approaches, we seek to generate a robust comparative genomic map of the harpy eagle relative to the putative ancestral avian karyotype and to better assess the evolutionary significance of shared chromosomal breakpoints within Accipitriformes.

## 2. Materials and Methods

### 2.1. Chromosomes and Karyotyping

Fibroblasts cryopreserved in the Cell Bank of the Laboratory of Cytogenomics and Environmental Mutagenesis (SEAMB-IEC) were used in this study. These cells belong to a lineage established from dermal pulp of a female Harpy eagle (*Harpia harpyja*), maintained at the Zoobotanical Park of the Museu Paraense Emílio Goeldi, as approved by the Ethics Committee for Animal Use of the Federal University of Pará (CEUA-UFPA 170/2013). For the determination of chromosome number and morphology, standard Giemsa staining (5% in phosphate buffer, pH 6.8) was performed for 5 min.

Karyotype assembly used slides stained with conventional Giemsa staining, analyzed under a Leica DM 1000 microscope (Leica Microsystems, Wetzlar, Germany) with an oil immersion objective. Image capture and karyotyping were carried out using GenAsis software version 7.2.6.19509 (Applied Spectral Imaging, California, USA). Ten metaphases were examined to standardize the karyotype.

### 2.2. FISH Experiments

Two sets of chromosome-specific probes were employed for FISH experiments: one corresponding to *Gallus gallus* chromosomes, included pairs GGA1–10, GGA Z, and a pool containing two pairs, identified as GGA10 and GGA11; and the other derived from *Leucopternis albicollis*, corresponding to segments homologous to GGA1–6: LAL1, LAL2, LAL3, LAL4, LAL5, LAL6, LAL7, as well as LAL8 and LAL13. These probes were labeled with fluorescein or biotin. Associations between chromosomal segments homologous to different GGA chromosomes and not applied simultaneously were inferred based on the consistent mapping of hybridization signals across multiple well-spread metaphases. Probe assignments were established considering chromosome morphology, including relative size and centromere position, as well as comparisons with signal patterns obtained from additional probe hybridizations. The integration of these parameters allowed reliable identification of chromosomal homologies and reduced the likelihood of misinterpretation caused by chromosome overlap or spatial proximity of signals.

It is noteworthy that the pooled probe containing GGA10 and GGA11 was used only for the detection of the presence of sequences homologous to either of these microchromosomes. The discrimination between GGA10 and GGA11 was inferred based on (i) the absence or presence of hybridization with individual whole-chromosome probes from other GGA macrochromosomes, (ii) the size and morphology of the painted segment, and (iii) comparison with homology patterns reported in previous chromosome painting studies and available genome assembly data. Therefore, although the pooled probe does not allow direct distinction between GGA10 and GGA11 at the hybridization step, the attribution of specific segments was supported by multiple independent lines of evidence.

The experiments were conducted following the protocol described by de Oliveira et al. [1], with modifications. Slides were incubated in HCl and pepsin for 3 min, followed by sequential dehydration in 70%, 90%, and 100% ethanol for 2, 2, and 4 min, respectively. Subsequently, slides were incubated at 37 °C for 1 h and at 60 °C for 25 min. After dehydration, chromosome denaturation was carried out by incubating the slides in 70% formamide at 72 °C. Immediately thereafter, slides were immersed in cold 70% ethanol, followed by a second ethanol series (70%, 90%, and 100%) for 2, 2, and 4 min, respectively. After air-drying at room temperature, 15 μL of hybridization solution (hybridization buffer and probes), previously denatured for 10 min at 70 °C in a water bath, was added to each slide. Coverslips were applied, sealed with adhesive plastic, and the slides were incubated in a humid chamber at 37 °C for 72 h.

Post-hybridization washes included incubation in 2 × SSC and 1 × SSC for 5 min each (twice), followed by 1 × PBS. For probes labeled with biotin, an additional detection step was performed: 200 μL of detection solution (199.6 μL of 4 × SST and 0.4 μL of Streptavidin-CY3) was added to the preparations. After a 30 min incubation in a dark, humid chamber, slides were washed three times in 4 × SST for 5 min each and subsequently dehydrated in ethanol series (70%, 90%, and 100%, 2 min each). Finally, a drop of antifade solution containing DAPI was applied, the preparations were covered with coverslips and sealed.

FISH analyses were performed using a Zeiss Axio Imager 7.2 microscope (Zeiss, Jena, Germany) equipped with a fluorescence system and an HBO 100 mercury lamp. Digital image acquisition was performed with 63× or 100× Plan-Apochromat objectives. Signal detection (probes and DAPI) was conducted using filters specific to the emission spectrum of each fluorophore, with modulation via Axiovision 4.8 software (Zeiss, Jena, Germany). Images were captured using a CCD camera and processed in Axiovision 4.8, then transferred to a computer for contrast adjustment, layer visualization, and color correction using Adobe Photoshop 2025. At least ten metaphase spreads per probe were analyzed to confirm the hybridization signal patterns.

### 2.3. Homology Maps

Comparative chromosomal mapping was based on the analysis of the in situ hybridization signals obtained using chromosome-specific probes from *G. gallus* and *L. albicollis*. The positioning of some segments was confirmed using data from the Comparative Genome Viewer tool, available on the NCBI platform.

The correspondence of *L. albicollis* probes to *G. gallus* chromosomes followed the mapping proposed by de Oliveira et al. [4]. Additionally, the correspondence suggested by Furo et al. [15] was indicated, including three microchromosomes of *L. albicollis* not previously described, two mapping to regions of GGA1 and one to GGA3.

## 3. Results

**Figure 1:**
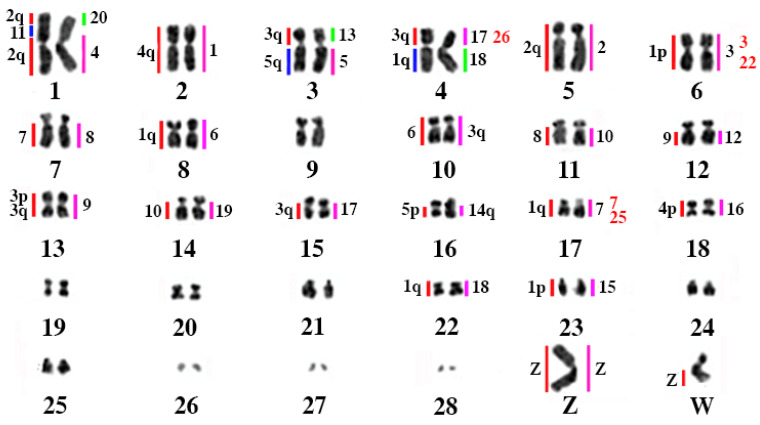
Homology map between *H. harpyja*, *G. gallus* (GGA), and *L. albicollis* (LAL). The correspondences between *H. harpyja* chromosomes and GGA are shown on the left (red/blue bars), while the correspondences with LAL are on the right (pink/green bars), following de Oliveira et al. [1]. For chromosome pairs HHA15, HHA17, and HHA23, an additional column indicating correspondences with LAL is highlighted in red, representing additional probe data from Furo et al. [11]. The chromosomal nomenclature adopted follows that of the NCBI, with the aim of facilitating future comparative studies, despite existing morphological discrepancies when compared to the original chromosome designations.

Chromosome-specific painting probes from *Gallus gallus* were used in hybridization experiments, targeting the first ten chromosome pairs (GGA1–10), in addition to a pooled probe containing GGA10 and GGA11. Chromosome painting probes from *Leucopternis albicollis* were also applied, covering segments homologous to GGA1–GGA7, both to validate the results and to identify potential chromosomal breakpoints shared between *Harpia harpyja* and this species. Table 1 provides a comparative overview of the results obtained in the present study, previous findings based on chromosome painting [6], and genome assembly data derived from whole-genome sequencing [14]. The comparative homology map, based on the chromosome painting results from this study, is presented in Figure 1.

**Figure 2:**
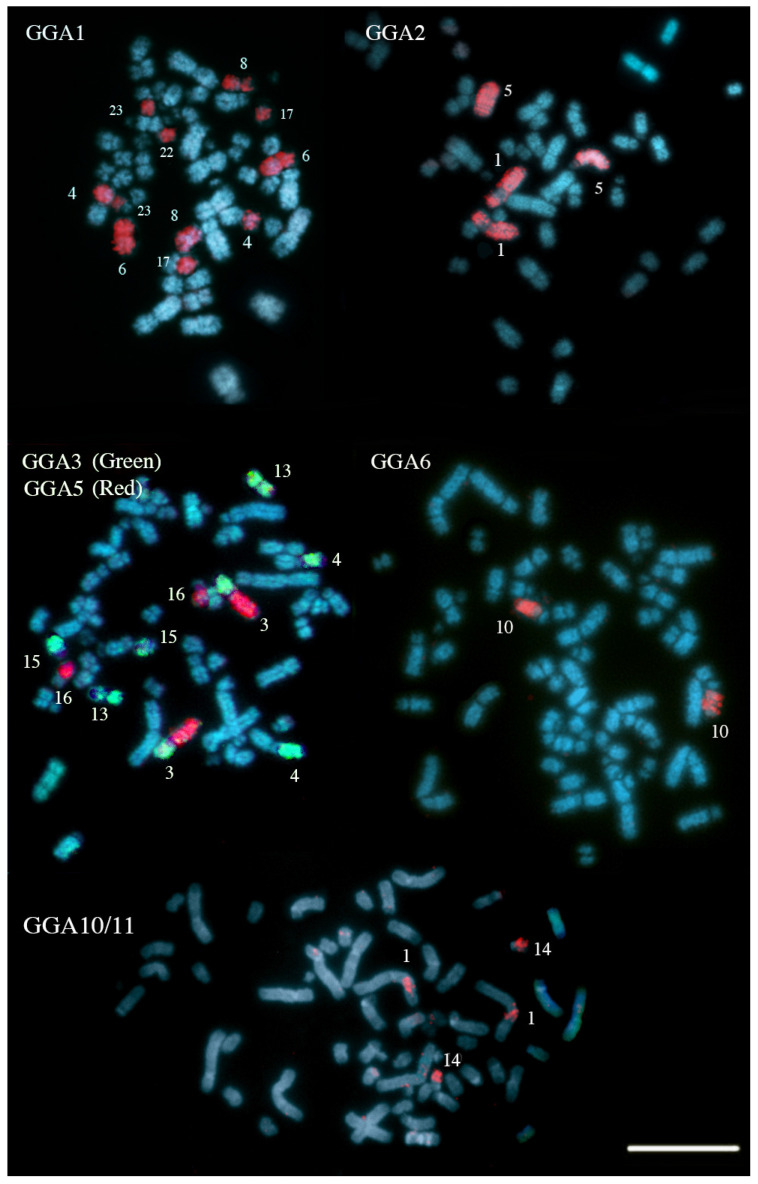
Representative results of fluorescent in situ hybridization (FISH) experiments using chromosome-specific probes from *G. gallus* (GGA) on metaphase chromosomes of *H. harpyja*. The probe(s) applied in each metaphase are indicated in the upper left corner of the respective image. Harpy eagle chromosome pairs showing hybridization numbered beside each chromosome. (Scale bar: 10 μm).

**Figure 3:**
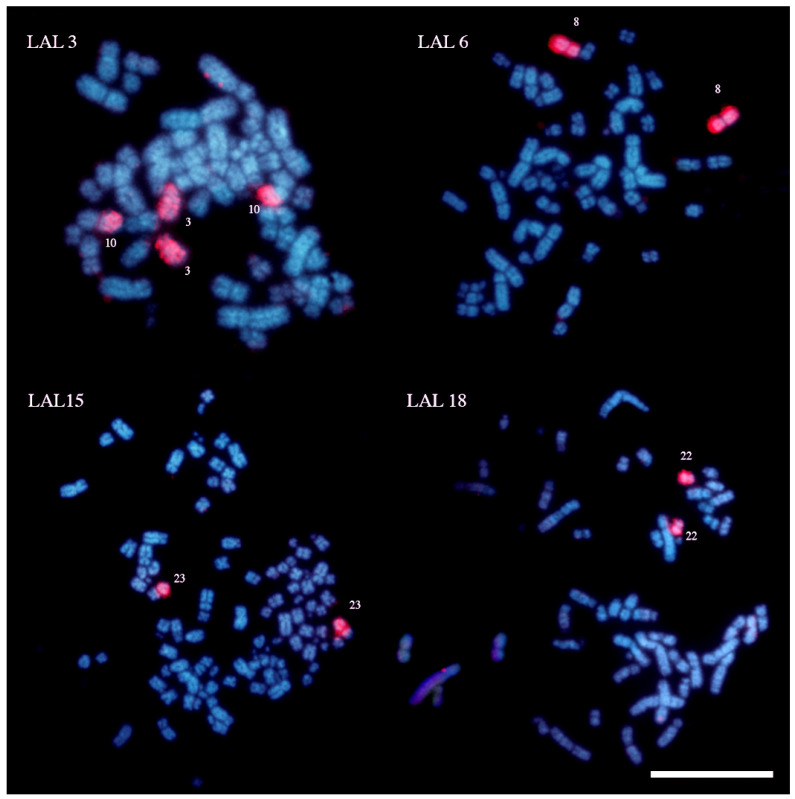
Representative results of fluorescent in situ hybridization (FISH) experiments using chromosome-specific probes from *L. albicollis* (LLA) on metaphase chromosomes of *H. harpyja*. The probes are indicated in the upper left corner of the respective image. Harpy eagle chromosome pairs exhibiting hybridization signals are labeled with numbers alongside each chromosome. The selected LLA probes correspond to *G. gallus* chromosome GGA1, except for LLA3, which hybridizes to a segment of GGA1p and to the pair corresponding to GGA6. (Scale bar: 10 μm).

Figure 2 and Figure 3 display representative FISH results obtained using *G. gallus* and *L. albicollis* chromosome-specific probes, respectively. The analysis of synteny among *H. harpyja*, *G. gallus*, and *L. albicollis* revealed the presence of several shared chromosomal breakpoints between the two Accipitriform species. Some of these are considered chromosomal breakpoint hotspots, particularly those occurring in the centromeric region of the ancestral chromosomes (PAK): putative ancestral avian karyotype. For instance, HHA6 corresponds to PAK1p. However, additional interstitial breakpoints also appear to be shared between *H. harpyja* and *L. albicollis*. In *L. albicollis*, two additional breakpoints occurred within PAK1p, producing three distinct segments (LAL3, LAL15, and LAL22), whereas in *H. harpyja*, PAK1p underwent a second fission event resulting in the formation of chromosome pair HHA23 (corresponding to LAL22). The long arm of PAK1 (PAK1q, represented by GGA1q) underwent extensive rearrangement in *H. harpyja*, giving rise to five different pairs: HHA1q, HHA8, HHA17, HHA22, and HHA23. These breakpoints were also identified in *L. albicollis*, which additionally exhibited a further fission in the region corresponding to HHA17, producing two distinct chromosomes in that species [15].

Regarding the second ancestral chromosome pair (corresponding to GGA2), a centric fission gave rise to HHA1 (PAK2q) and HHA1 (PAK2p), a rearrangement also shared with *L. albicollis*. However, the GGA2q-derived segment in *L. albicollis* underwent an additional breakpoint. Interestingly, in the harpy eagle, this breakpoint region appears to have undergone an insertion event involving the microchromosome GGA11.

The third ancestral pair, corresponding to GGA3, experienced four distinct breakpoints. The resulting segments were subsequently involved in fusion events, forming chromosomes such as HHA3 and HHA4 (with the short arms derived from PAK3 segments), as well as HHA13 and HHA15. These breakpoints were also shared with *L. albicollis*. GGA5 is represented by two separate chromosomes due to a centric fission: the segment corresponding to GGA5q is fused with a GGA3-derived segment to form HHA3, while GGA5p corresponds to HHA16. GGA4 arms are segregated into two separate chromosomes in the harpy eagle—HHA2 (GGA4q, and PAK4) and HHA18 (GGA4p, and PAK11)—which reflects the ancestral avian condition.

The remaining macrochromosomes of *Gallus gallus* (GGA6–GGA10) retained syntenic integrity in Harpia, appearing as entire chromosomes or chromosome segments without evidence of chromosomal breakage.

## 4. Discussion

The results obtained from the hybridization of individual chromosome-specific probes of *Gallus gallus* and *Leucopternis albicollis* in *Harpia harpyja* showed few discrepancies compared to previously published data based on chromosome painting [5] and Next-Generation Sequencing (NGS) data [14]. Our findings were largely consistent with those presented in the earlier study [6], except for two key differences: our results revealed that GGA1 is represented by six distinct segments, rather than five, and indicated a GGA1/GGA3 association that was not identified in the earlier work. Additionally, we observed an insertion of GGA11 in the interstitial region of the short arm of HHA1, whereas in the previous study, this insertion was mapped to pair HHA2.

These discrepancies may be attributed to methodological limitations, as the authors of the earlier study inferred homology for GGA1 to GGA6 and GGA Z in the harpy eagle using a seven-colour pool. This approach hindered accurate interpretation due to reduced color discrimination. Moreover, the metaphase chromosomes obtained from lymphocyte cultures were notably short, further complicating morphological assessments.

A methodological consideration of the present study is the use of a pooled probe for GGA10 and GGA11, which does not permit direct discrimination between these two chromosomes during hybridization. Consequently, the assignment of the interstitial insertion observed in HHA1p to GGA11 was based on comparative evidence from other GGA probes, previous chromosome painting data, and genome assembly annotations. Although this approach provides strong indirect support, the precise identity of this inserted segment will require confirmation using chromosome-specific BAC probes or individually labeled GGA10 and GGA11 probes.

Regarding the NGS data, although most findings on the number of homologous segments for each GGA pair were concordant with ours, some differences were noted, particularly concerning the alignment positions of key macrochromosomal segments. These differences can be ascribed to inherent limitations of genome sequencing, especially its inability to effectively resolve regions rich in repetitive sequences. Such repetitive regions often lead to errors in chromosomal size estimation and synteny reconstruction. The presence of long repetitive sequences remains a major obstacle in generating complete genome assemblies. Despite the vast genomic datasets generated by NGS, many genomes remain incomplete due to challenges in assembling repetitive elements [16,17,18]. Short reads produced by NGS platforms typically cannot span long repeats, and the high similarity among repeated sequences often leads to misassembly and collapsed contigs. While continued advances in bioinformatics seek to address these issues [17], accurately reconstructing long repetitive regions still exceeds current capabilities. Additionally, repetitive DNA segments are often lost during cloning steps in genome assembly workflows [18,19].

To reconcile discrepancies between chromosome-level sequencing data and morphology-based classifications derived from cytogenetic analyses, we adopted the nomenclature provided by the NCBI database [14], as summarized in Table 1. Apart from this issue, the principal disagreement between our results and the NCBI annotations concerns the identity of the sequence inserted in the interstitial region of HHA1p. While our cytogenetic data indicate the presence of GGA11 in this region, sequencing-based annotations assign this segment to GGA20. Furthermore, whole-chromosome probes for GGA Z and LLA Z hybridized along the entire length of HHA Z and partially on HHA W, providing no support for the putative GGA12/GGA Z fusion reported in the NCBI database.

Although definitive characterization of sex chromosome systems is traditionally supported by meiotic analyses, such approaches require access to gonadal tissues. In large and threatened raptor species such as *H. harpyja*, the collection of meiotic material would typically involve euthanasia, which is ethically and legally restricted and therefore rarely feasible. Consequently, direct meiotic investigation of sex chromosome behavior in this species is unlikely to be achievable in practice. In this context, integrative approaches based on cytogenomics, chromosome painting, and chromosome-level genome assemblies represent realistic and increasingly accepted alternatives for investigating sex chromosome organization and testing hypotheses regarding the presence of complex sex chromosome systems. Therefore, our conclusions should be interpreted within the framework of currently accessible methodologies, which provide strong evidence against the previously proposed autosome–Z translocation, while acknowledging the inherent limitations associated with the study of endangered species.

An additional source of uncertainty is the possibility that the GGA11 whole-chromosome probe generated by flow sorting actually corresponds to GGA12 in the sequencing data, as probe nomenclature was originally based solely on the size and morphology of the painted chromosomes. Nevertheless, a discrepancy would remain, since this probe hybridized to an interstitial region of a large chromosome pair. Consistently, hybridization with the GGA2 whole-chromosome probe revealed a gap in the short arm of HHA1 (Figure 2), further supporting this interpretation. Future analyses using BAC probes will be necessary to confirm or refute these cytogenomic interpretations with greater confidence.

The inclusion of *L. albicollis* probes alongside *Gallus* probes represented a significant methodological advancement, providing additional cytogenetic information. Hybridization with *L. albicollis* probes revealed that species from distinct subfamilies share breakpoints in ancestral macrochromosomes, suggesting these events occurred early in accipitrid evolution, prior to the divergence of the Buteoninae and Harpiinae subfamilies. Furthermore, the observation that these breakpoints are also shared with the neotropical Aquilinae species *Spizaetus tyrannus*, which exhibits the same fission pattern in ancestral pairs GGA1, GGA2, and GGA3 [20], and conserved breakpoints across species based on *L. albicollis* probes, further supports the hypothesis that cytogenomic reorganization in Accipitriformes occurred early, before diversification of these clades.

Despite broad agreement on the number of families and subfamilies within Accipitriformes, substantial discrepancies persist among proposed phylogenetic hypotheses, and several inferred groupings appear to be non-monophyletic [21]. In particular, major phylogenetic frameworks differ in their placement of the three subfamilies within the order. The most recent phylogeny recovers Harpiinae as the sister group to the Buteoninae + Accipitrinae clade [21], whereas earlier studies associated Harpiinae either with Aquilinae alone [22,23] or with a more inclusive clade comprising Aquilinae, Buteoninae, and Accipitrinae [24].

Our cytogenomic data indicate that breakpoints involving ancestral chromosomes corresponding to the first *G. gallus* (GGA) pairs are shared among the harpy eagle, representatives of Buteoninae, and *S. tyrannus*, a Neotropical member of Aquilinae. This pattern suggests that these chromosomal fissions originated in a common ancestor of the three lineages. Accordingly, our findings are more consistent with the hypothesis proposed by Lerner et al. [24], which places Harpiinae as the sister group to a clade encompassing Aquilinae, Buteoninae, and Accipitrinae.

In a previous cladistic analysis that mapped chromosomal rearrangements and syntenic associations onto a consensus molecular phylogeny, the harpy eagle exhibited unstable placements across the most parsimonious trees, largely due to the limited comparative chromosome painting data available at the time [13]. The incorporation of the new data presented here, however, would be unlikely to resolve the position of this species, as most newly identified associations and breakpoints would represent autapomorphies relative to other taxa in the analysis. Notably, the chromosomal associations GGA2q/11, GGA1q/3q, and GGA3q/5q have not been reported in any other Accipitriformes to date. Consequently, a critical next step will be to determine whether these associations are also present in *Morphnus guianensis*, the sister species of the harpy eagle within the subfamily Harpiinae.

A limitation of the present study is that all cytogenetic analyses were performed using a single fibroblast cell line derived from one female individual. However, most chromosome rearrangements observed during the evolution of birds correspond to inversions [25,26,27], as well as in the examples of chromosome polymorphism [28,29,30]. Additionally, all previously published karyotypic descriptions for this species consistently report a diploid number of 2n = 56 and a highly similar chromosomal morphology [6,9]. These observations reinforce the interpretation of conserved syntenic organization in this species. Furthermore, the overall karyotypic organization observed here is largely consistent with previous chromosome painting results and with the available chromosome-scale genome assembly, supporting the interpretation that the major rearrangements described here likely represent species-level characteristics. In addition, the overall karyotypic organization observed here is largely concordant with previous chromosome painting results in *H. harpyja* and with the available chromosome-scale genome assembly, which supports the interpretation that the major rearrangements reported represent species-level characteristics. Future analyses including additional individuals, ideally from both sexes, will be essential to assess the extent of intraspecific variation and to confirm the stability of these rearrangements.

## 5. Conclusions

The integration of classical cytogenetic techniques with chromosome-specific probes from *Gallus gallus* and *Leucopternis albicollis*, along with recent genomic data, has provided a more comprehensive and refined understanding of the complex karyotypic evolution in *Harpia harpyja*. The identification of previously undetected rearrangements, such as novel fissions, fusions, and interstitial insertions, highlights the importance of employing complementary methodologies to overcome the limitations inherent in both chromosome painting and genome sequencing.

The conservation of chromosomal breakpoints across distinct subfamilies further supports the hypothesis of an early and shared chromosomal reorganization event in the evolutionary history of Accipitriformes. Moreover, the congruence of these cytogenomic patterns with certain phylogenetic frameworks lends additional support to specific evolutionary relationships within the group. Collectively, these findings not only elucidate the mechanisms driving chromosomal evolution in birds of prey but also contribute to the broader understanding of avian genome architecture and its evolutionary dynamics. Future studies employing BAC probes will be crucial for resolving remaining uncertainties and advancing the field of avian cytogenomics.

## Figures and Tables

**Table 1:** Comparative homology of *G. gallus* chromosomes with *H. harpyja*. Based on genome sequencing data, previous chromosome painting reports, and results of the present study. Nomenclature of the segments were adapted to follow the NCBI database [14].

*Gallus gallus*	Homology with *H. harpyja* (Sequencing Data)	Previous Reports [5]	This Study
GGA1	Six segments (4q, 6, 8, 17, 22 and 23)	Five segments (5, 6, 19, 21, and 24)	Six segments (4q, 6, 8, 17, 22, and 23)
GGA2	Two pairs (1 and 5), with GGA20 inserted in HHA1p	Two pairs (1 and 3)	Two pairs (1 and 5), with GGA11 inserted in HHA1p
GGA3	Four segments (3p, 4p, 13, and 15)	Four segments (2p, 10, 18, and 23)	Four segments (3p, 4p, 13, and 15)
GGA4	Two pairs (2 and 18)	Two pairs (4 and 14)	Two pairs (2 and 18)
GGA5	Two segments (3q and 16q)	Two segments (2q and 20)	Two segments (3q and 16q)
GGA6	One pair (10)	One pair (8)	One pair (8)
GGA7	One pair (7)	-	One pair (7)
GGA8	One segment (11q)	-	One segment (11q)
GGA9	One segment (12q)	-	One segment (11q)
GGA11	One segment (9p)	-	One segment (1p)
GGA Z	Z, W, and 12	Z and W	Z and W

## Data Availability

All data from this study are available in the manuscript. In addition, other information can be obtained request from the corresponding author.
